# Cementless versus hybrid fixation in primary total knee arthroplasty: a propensity score-matched analysis of mid-term outcomes

**DOI:** 10.1186/s42836-026-00399-2

**Published:** 2026-07-01

**Authors:** Juan Miguel Gómez-Palomo, Vicente de la Varga-Cebrián, Ana Martínez-Crespo, Carmen Tara-Abad, Claudia Aguilar-López, Plácido Zamora-Navas

**Affiliations:** 1 Department of Orthopedic Surgery and Traumatology, Virgen de La Victoria University Hospital, Campus Teatinos, S/N, 29010 Málaga, Spain; 2 Biomedical Research Institute of Málaga (IBIMA), Doctor Miguel Díaz Recio Street, 28, 29010 Málaga, Spain; 3 Faculty of Medicine, University of Málaga, Louis Pasteur Boulevard, 32, 29071 Málaga, Spain

**Keywords:** Total knee arthroplasty, Cementless fixation, Hybrid fixation, Complications, Radiolucent lines, Functional outcomes

## Abstract

**Purpose:**

Total knee arthroplasty (TKA) is an effective treatment for advanced gonarthrosis. This retrospective study compares cementless versus hybrid fixation in TKA, an under-investigated comparison in the literature, to describe mid-term clinical, radiographic, and perioperative outcomes between both fixation strategies.

**Methods:**

We conducted a retrospective cohort study of 166 patients (age 50–70, adequate bone quality) undergoing primary cruciate-retaining TKA from November 2019 to May 2024. Fixation type was selected by the treating surgeons according to intraoperative assessment of tibial bone stock and press-fit stability. Patients were divided into two groups based on fixation: uncemented (cementless) vs. hybrid (cementless femoral, cemented tibial). Clinical (pain, function) and radiographic outcomes, perioperative complications, and surgical time were compared. Functional outcomes were assessed with Hospital for Special Surgery (HSS) and WOMAC scores; quality of life with the EQ-5D. A propensity score-matched analysis (1:1) was additionally performed to mitigate measured baseline confounding, matching on age, sex, body mass index (BMI), Charlson comorbidity index and preoperative HSS score.

**Results:**

The mean follow-up was 36.8 ± 18.7 months. Uncemented fixation was associated with significantly shorter surgical time (65.2 ± 12.4 vs. 78.6 ± 15.1 min; *p* = 0.032), a modestly greater postoperative pain reduction on the VAS (7.25 ± 2.02 vs. 6.41 ± 1.94 points; *p* = 0.012), and a lower observed rate of late complications (1.2% vs. 10.8%; *p* = 0.028) compared to hybrid. The incidence of aseptic loosening was lower in the uncemented group (0.0% vs. 3.6%; *p* = 0.029), as was the presence of radiolucent lines (6.0% vs. 18.1%; *p* = 0.032). No significant differences were found in postoperative range of motion, HSS score improvements, WOMAC, or EQ-5D index between groups. The propensity-matched sub-analysis (*n* = 150) yielded directionally consistent findings, although these analyses reduce but do not eliminate residual confounding and remain underpowered for rare outcomes.

**Conclusion:**

Cementless TKA was associated with comparable mid-term functional outcomes to hybrid fixation, together with shorter operative time and lower observed rates of radiolucent lines and late complications. The difference in pain reduction was statistically significant but modest, and radiographic findings and rare events should be viewed as hypothesis-generating rather than as evidence of superior fixation performance or long-term implant durability. These findings suggest that cementless fixation may represent a reasonable alternative to hybrid fixation in appropriately selected patients, although longer-term prospective comparative studies are required to confirm implant survivorship and clarify the clinical relevance of these observations.

## Introduction

Total knee arthroplasty (TKA) is a widely accepted procedure for end-stage gonarthrosis. Cemented fixation has traditionally been considered the gold standard due to its immediate mechanical stability, ease of use in osteoporotic bone, and its role in delivering local antibiotics to prevent infection. Additionally, the cement mantle acts as a barrier to polyethylene wear particles, potentially reducing osteolysis [1, 2].

However, demographic and lifestyle changes, including increased life expectancy, obesity, and a more active elderly population, have driven interest in alternative fixation strategies [3]. Younger TKA patients exhibit higher revision rates, mainly due to aseptic loosening, and obese patients (BMI > 35) face a two-fold increased risk of early failure [4–6].

In this context, cementless fixation has gained attention as a means to enhance implant longevity. Unlike mechanical cemented fixation, cementless implants rely on biological fixation through bone ingrowth into porous surfaces, theoretically providing long-term stability and allowing bone remodeling. This approach may be particularly advantageous in patients with good bone quality and high functional demands [7].

Initial cementless TKA designs were met with skepticism due to poor clinical outcomes attributed to factors like non-crosslinked polyethylene, inadequate porous coatings, and flawed component designs, leading to high tibial loosening rates [7, 8].

With recent design improvements, modern cementless TKA now offers promising results. The current challenge lies in identifying patient subgroups who may benefit most and in assessing long-term outcomes. While numerous studies have compared fully cemented and cementless TKAs, data specifically contrasting cementless with hybrid fixation (cementless femoral component combined with a cemented tibial component) are limited. This less commonly studied comparison is clinically relevant, as hybrid TKA is an intermediate strategy used by some surgeons.

This study aims to compare the clinical, radiographic, and surgical outcomes between cementless and hybrid fixation in primary TKA. We hypothesized that cementless fixation would provide clinical and radiographic outcomes comparable to hybrid fixation, with potential advantages in operative efficiency and without an increased complication rate in patients under 70 years old with adequate bone quality.

## Materials and methods

### Study design and ethical approval

This was a retrospective cohort study conducted at the Department of Orthopedic Surgery of a high-volume arthroplasty center. The study adhered to the principles of the Declaration of Helsinki (2013) and was approved by the Provincial Research Ethics Committee of Málaga, Spain (reference code CEI-24_2020; SICEIA-2024-001425). Informed consent for participation and data use was obtained from all patients.

### Study population

Between November 2019 and May 2024, 174 patients who underwent primary TKA using the Triathlon® cruciate-retaining (CR) prosthesis (Stryker®) were screened. After excluding eight patients lost to follow-up (4.6%), 166 patients met the inclusion criteria and were included in the final analysis. Patients were classified into two cohorts based on the fixation technique used:

- Cementless group (*n* = 83): Both femoral and tibial components were implanted without cement, relying on porous-coated surfaces for biological fixation via osseointegration.
- Hybrid group (*n* = 83): The femoral component was implanted press-fit (uncemented), while the tibial component was cemented using polymethylmethacrylate (PMMA).

#### Inclusion criteria

To ensure homogeneity and reduce confounding, strict inclusion criteria were applied:

- Age between 50 and 70 years at the time of surgery
- Diagnosis of primary osteoarthritis (Kellgren–Lawrence grade III or IV)
- Use of the Triathlon® CR TKA system (available in both cementless and hybrid versions)
- Body mass index (BMI) ≤ 40 kg/m^2^
- Minimum follow-up of 24 months postoperatively
- Availability of complete pre- and post-operative clinical and radiological data

#### Exclusion criteria

Patients were excluded if they met any of the following:

- Moderate to severe osteoporosis (history of fragility fracture or densitometric confirmation)
- Inflammatory arthropathy (e.g., rheumatoid arthritis, psoriatic arthritis)
- Prior surgery on the affected knee (osteotomy, ligament reconstruction, intra-articular fracture with sequelae)
- Severe coronal deformity > 15° varus/valgus or flexion contracture > 20°
- Neuromuscular disorders affecting the knee (e.g., Parkinson’s, multiple sclerosis, advanced diabetic neuropathy)
- Active smokers (> 20 cigarettes/day)
- Active infection at time of surgery (systemic or local)
- Refusal to provide informed consent

### Surgical technique and perioperative protocol

All TKAs were performed by the same team of senior knee arthroplasty surgeons, rather than by a single surgeon, with extensive experience in primary and revision arthroplasty at a high-volume center.

A standard medial parapatellar approach was used in all cases, preserving the extensor mechanism. Key perioperative protocols included:

- Antibiotic prophylaxis: 2 g intravenous cefazolin 30 min preoperatively (or 1 g vancomycin for beta-lactam allergy).
- Anesthesia: Spinal anesthesia in all patients, with sedation as required.
- Postoperative analgesia: The same standardized postoperative analgesic regimen was used in both groups, consisting of paracetamol and tramadol for all patients, except in cases of allergy or contraindication, where treatment was modified accordingly.
- Thromboprophylaxis: All patients underwent a preoperative anesthetic assessment, during which thromboprophylaxis was reviewed according to their individual risk profile. In most cases, the standard institutional regimen consisted of low molecular weight heparin (enoxaparin 40 mg daily), starting 12 h postoperatively and continued for 30 days; however, prophylaxis was adjusted when clinically indicated based on patient-specific thromboembolic and bleeding risks.
- Implant specifics: In the hybrid group, only the tibial component was cemented with standard PMMA cement; in the cementless group, both femoral and tibial components had highly porous coatings to facilitate bone ingrowth.
- Patellar management: Patella was selectively resurfaced based on intraoperative cartilage assessment and surgeon judgment.
- Rehabilitation: The same standardized postoperative rehabilitation protocol was used in both groups, with full weight-bearing of the operated limb permitted from the immediate postoperative period and early mobilization beginning on postoperative day 1 under physiotherapy supervision.

### Data collection and variables

Data were collected prospectively in an institutional registry and reviewed retrospectively for this study. Baseline demographic and clinical characteristics recorded were age, sex, BMI, comorbidities (Charlson Comorbidity Index), and lifestyle factors (tobacco and alcohol use). Preoperative knee function was assessed by the Hospital for Special Surgery (HSS) score. Operative details (side of surgery, patellar resurfacing) were noted.

Postoperative outcomes included:

- Surgical time (minutes)
- Pain assessed by the Visual Analog Scale (VAS)
- Functional outcomes via HSS and WOMAC scores at final follow-up
- Quality of life via EuroQol 5-Dimension (EQ-5D) index
- Radiographic assessment of radiolucent lines and fixation-interface integrity, evaluated according to the Modern Knee Society Radiographic Evaluation System
- Complications (local: periprosthetic fracture, infection, aseptic loosening, stiffness; systemic: thromboembolism, myocardial infarction, pneumonia, etc.)
- Any reoperations, readmissions, or all-cause mortality

Standardized anteroposterior and lateral radiographs were obtained at 1 month, 3 months, and annually. Radiolucent lines were assessed using the Modern Knee Society Radiographic Evaluation System and recorded according to the predefined femoral and tibial zones. They were defined as linear radiolucencies > 1 mm at the bone–implant interface on follow-up radiographs and were further assessed for progression over time on serial imaging.

### Bias control and blinding

The fixation strategy was not randomized. The choice of cementless or hybrid fixation followed institutional practice and was surgeon-directed, based on intraoperative assessment of tibial bone stock and anticipated press-fit stability of the tibial component. This assessment was not based on a standardized objective bone-quality measure, such as routine DEXA/BMD testing or a prespecified intraoperative bone-quality scoring system; therefore, fixation allocation remained susceptible to confounding by indication. Patients meeting the inclusion criteria typically received cementless fixation if tibial bone stock was deemed strong and press-fit stability was considered adequate, whereas hybrid fixation was selected when there were intraoperative concerns regarding tibial fixation stability or during the early phase of cementless implant adoption at our center.

Data collection, including radiographic outcome abstraction for study purposes, was performed by an independent evaluator who was not involved in the surgical procedures and was blinded to the type of fixation. Formal inter- and intraobserver reliability analyses were performed in a representative subset of radiographs, demonstrating high agreement (interobserver kappa = 0.82; intraobserver kappa = 0.88), supporting the reliability of the radiographic assessment. Patients lost to follow-up were documented, with losses evenly distributed between groups. A multivariate analysis (logistic regression) was conducted to account for potential confounders (age, sex, and Charlson index). In addition, a propensity score matching analysis was performed post hoc to further address selection bias (see Statistical Analysis).

### Statistical analysis

Data were analyzed using SPSS® Statistics v25 (IBM Corp., Armonk, NY, USA). Continuous variables were tested for normality (Kolmogorov–Smirnov test). Most did not follow a normal distribution, so comparisons between groups used non-parametric tests (Mann–Whitney U). Categorical variables were compared with Chi-square or Fisher’s exact test, as appropriate. Statistical significance was set at *p* < 0.05 (two-tailed). For the main between-group postoperative comparisons, mean differences with 95% confidence intervals were calculated for continuous outcomes, and relative risks (RR) with 95% confidence intervals (CI) were calculated for categorical outcomes. A multivariate logistic regression was performed to adjust for potential confounders.

Because implant selection was surgeon-directed in this retrospective clinical cohort, PSM was used as a post hoc sensitivity analysis to improve balance in measured baseline covariates and to mitigate—but not eliminate—confounding by indication. Randomization was not feasible because treatment allocation had already occurred as part of routine clinical practice before the study was designed. Accordingly, the matched analysis was intended to assess the consistency of the observed associations rather than to establish causal effects.

The propensity score for receiving cementless fixation was estimated for each case using a logistic regression model including baseline covariates: age, sex, BMI, Charlson comorbidity index, and preoperative HSS score. One-to-one nearest-neighbor matching without replacement was carried out with a caliper of 0.2 of the propensity score standard deviation, chosen to reduce poor matches while preserving an adequate matched sample size, yielding 75 matched pairs (150 patients total). Balance between matched groups was assessed by comparing baseline covariates after matching, with no significant differences identified. Post-matching comparisons were performed using paired statistical tests: Wilcoxon signed-rank tests for continuous outcomes and McNemar’s test for paired categorical data. However, PSM could not account for unmeasured variables such as activity level, objective bone mineral density, deformity severity within the included range, temporal adoption effects, or surgeon-specific intraoperative decision factors.

To address multiplicity across key secondary postoperative between-group comparisons, a sensitivity analysis was performed using the Benjamini–Hochberg procedure to control the false discovery rate. Adjusted p-values were calculated for the prespecified family of secondary outcomes related to operative efficiency and implant-related safety/radiographic performance (surgical time, VAS pain reduction, late complications, aseptic loosening, and radiolucent lines), while raw *p*-values were also reported for transparency.

## Results

**Fig. 1:**
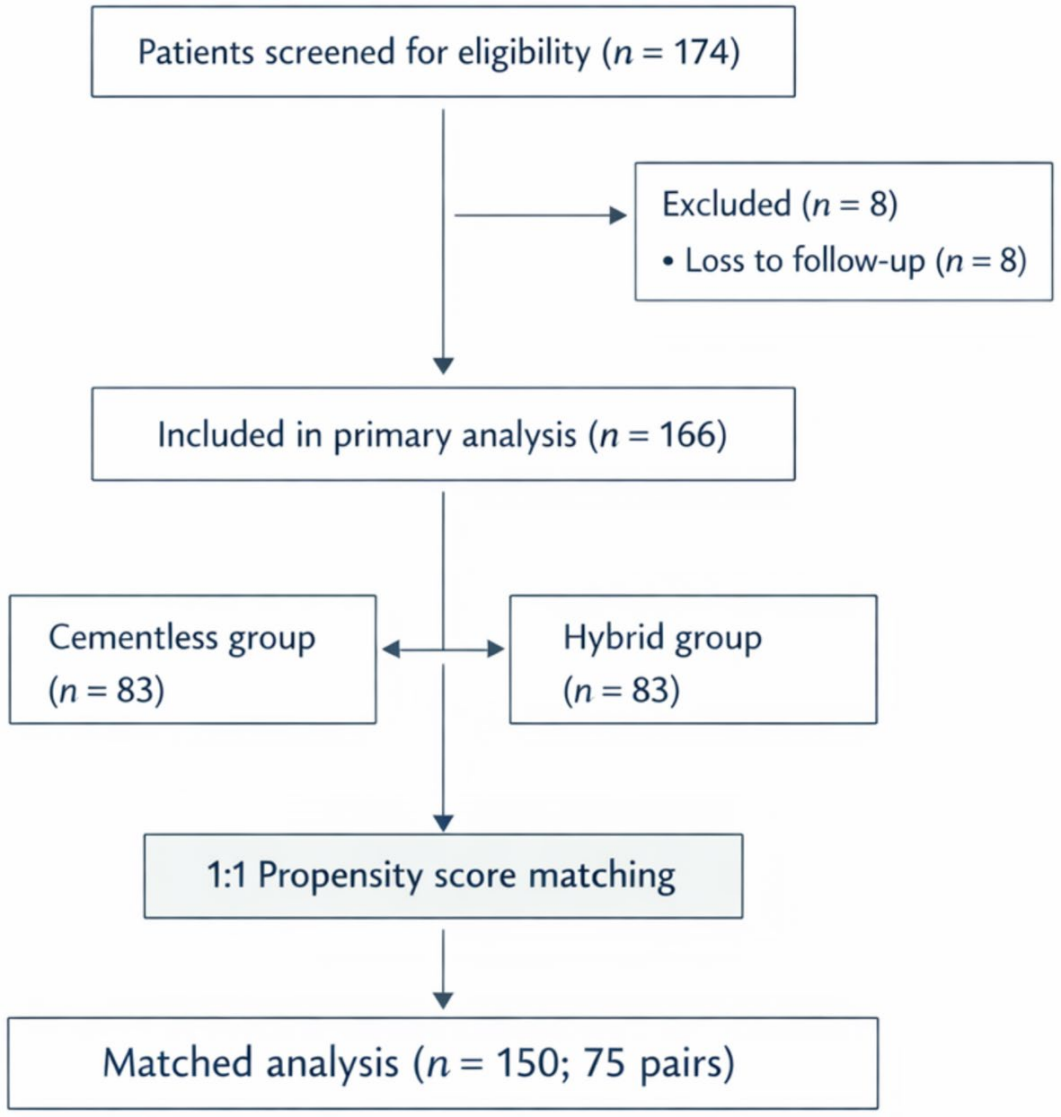
Flow diagram of patient selection and propensity score matching. Of 174 screened patients, 8 were excluded due to loss to follow-up, leaving 166 patients for the primary analysis (83 cementless and 83 hybrid). After 1:1 propensity score matching, 150 patients (75 pairs) were included in the matched analysis

A total of 166 patients met the inclusion criteria and were analyzed, with 83 in the cementless fixation group and 83 in the hybrid fixation group. The overall mean follow-up period was 36.8 ± 18.7 months. Eight patients (4.6%) were lost to follow-up (4 from each group) and were excluded from analysis. A flow diagram summarizing patient screening, exclusions, final cohort allocation, and the propensity-matched sub-analysis is presented in Fig. 1.

### Baseline characteristics

Baseline demographic and clinical characteristics were comparable between groups (Table 1). There were no significant differences in sex distribution (49.4% male in hybrid vs. 51.8% in cementless; *p* = 0.232) or laterality (left vs. right knee, *p* = 0.877). Patellar resurfacing was performed in 34% of the cementless group and 36% of the hybrid group, indicating a very similar distribution between groups. The mean age was 62.3 ± 5.7 years (hybrid) vs. 61.8 ± 5.4 (cementless; *p* = 0.210). Mean BMI was 30.1 ± 4.4 (hybrid) vs. 30.4 ± 4.2 (cementless; *p* = 0.105). Charlson Comorbidity Index was 2.4 ± 1.3 vs. 2.3 ± 1.2 (*p* = 0.306). The prevalence of individual comorbid conditions (hypertension, diabetes, pulmonary disease, etc.) was similar between the two cohorts, with no significant differences.

**Table 1:** Baseline characteristics of the study population (unmatched cohort)

Variable	Hybrid Fixation (*n* = 83)	Cementless Fixation (*n* = 83)	*p*-value
Sex (Male/Female)	41 (49.4%)/42 (50.6%)	43 (51.8%)/40 (48.2%)	0.232
Laterality (Left/Right)	40 (48.2%)/43 (51.8%)	39 (47.0%)/44 (53.0%)	0.877
Age (years), mean ± SD	62.3 ± 5.7	61.8 ± 5.4	0.210
BMI (kg/m^2^), mean ± SD	30.1 ± 4.4	30.4 ± 4.2	0.105
Charlson Comorbidity Index	2.4 ± 1.3	2.3 ± 1.2	0.306

Categorical variables compared via Chi-square/Fisher’s exact test; continuous variables via Mann–Whitney U test (non-normal distributions)

### Surgical and clinical outcomes

Several between-group differences were observed in postoperative outcomes (Table 2). The mean surgical time was significantly shorter for cementless TKAs (65.2 ± 12.4 min) compared with hybrid TKAs (78.6 ± 15.1 min), corresponding to a mean difference of − 13.4 min (95% CI, − 17.6 to − 9.2; *p* = 0.032).

**Table 2:** Postoperative clinical and radiological outcomes (unmatched cohort)

Outcome	Hybrid Fixation (*n* = 83)	Cementless Fixation (*n* = 83)	*p*-value
Surgical time (min), mean ± SD	78.6 ± 15.1	65.2 ± 12.4	0.032
VAS pain reduction (0–10), mean ± SD	6.41 ± 1.94	7.25 ± 2.02	0.012
Late complications, *n* (%)	9 (10.8%)	1 (1.2%)	0.028
Aseptic loosening, *n* (%)	3 (3.6%)	0 (0.0%)	0.029
Radiolucent lines, *n* (%)	15 (18.1%)	5 (6.0%)	0.032
Postoperative ROM (degrees)	97.89 ± 12.36	97.93 ± 9.84	0.760
HSS score improvement, mean ± SD	41.15 ± 16.90	41.12 ± 10.99	0.599
EQ-5D index (final follow-up)	0.84 ± 0.09	0.86 ± 0.08	0.104

Continuous variables compared with the Mann–Whitney U test; categorical outcomes with Chi-square/Fisher’s test. “VAS pain reduction” denotes a decrease in VAS pain score from preoperative baseline to final follow-up

Postoperative pain improvement, measured as the decrease in VAS from baseline to final follow-up, was modestly greater in the cementless cohort (mean VAS reduction 7.25 ± 2.02) than in the hybrid cohort (6.41 ± 1.94), corresponding to a mean difference of 0.84 points (95% CI, 0.23 to 1.45; *p* = 0.012). Although this between-group difference reached statistical significance, its magnitude was small, and its clinical relevance remains uncertain; therefore, it should be interpreted cautiously.

In terms of complications, the cementless group had a significantly lower incidence of late postoperative complications (defined as those occurring beyond 30 days). Only 1 patient (1.2%) in the cementless group experienced a late complication, versus 9 patients (10.8%) in the hybrid group, corresponding to a relative risk of 0.11 (95% CI, 0.01 to 0.86; *p* = 0.028). Late complications were analyzed as a composite endpoint comprising the predefined local and systemic postoperative adverse events listed in the Methods section. Because individual complication categories were infrequent, they were not compared separately between groups. Given the low absolute number of events, this composite outcome should be interpreted as exploratory and hypothesis-generating.

Aseptic loosening was documented in 3 cases (3.6%) in the hybrid group, whereas no cases occurred in the cementless group (*p* = 0.029). With only three events overall, this comparison was underpowered and statistically unstable; therefore, it should be interpreted cautiously and does not allow firm conclusions regarding fixation durability.

**Fig. 2:**
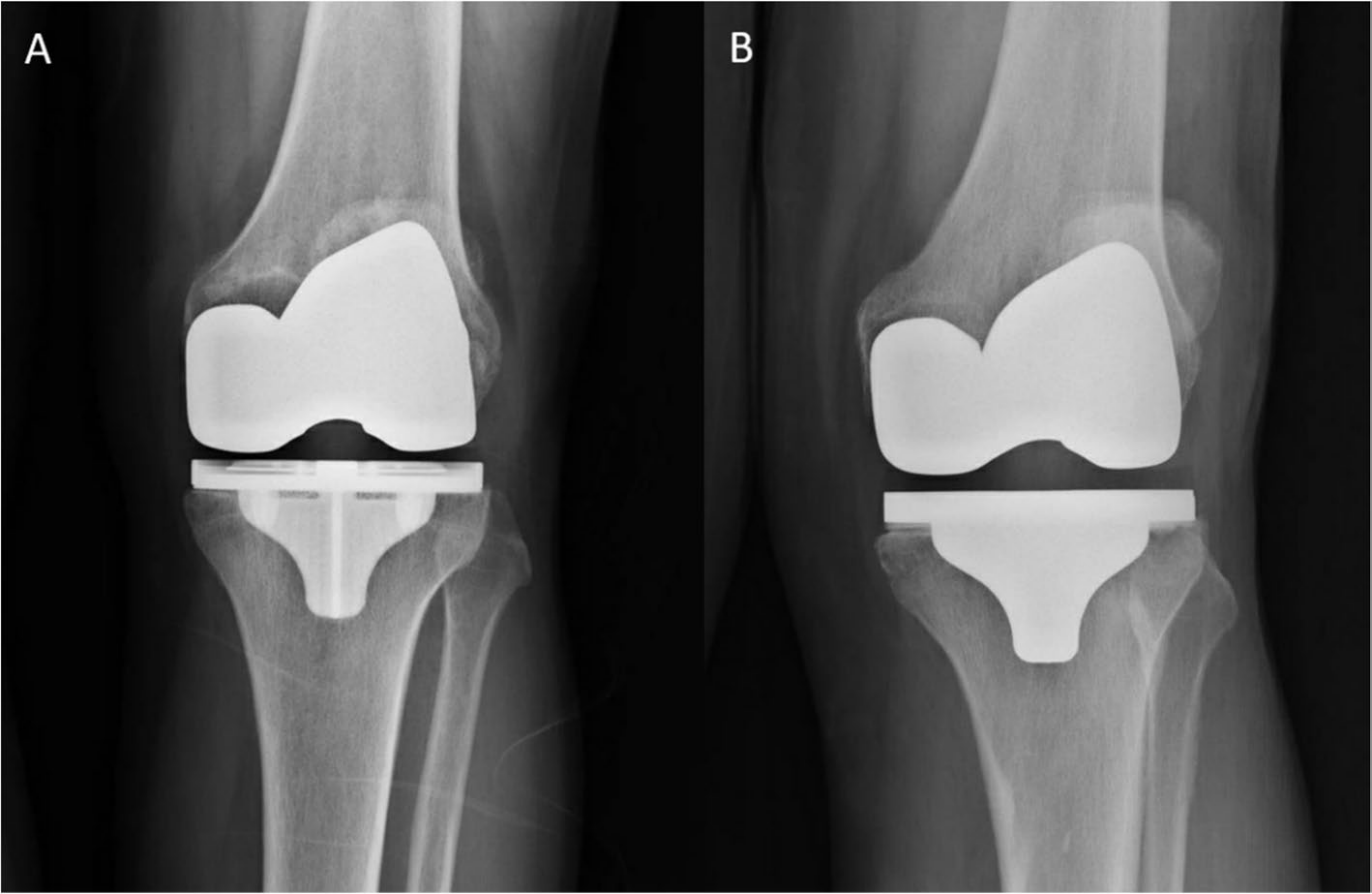
Representative anteroposterior postoperative radiographs of cementless (**A**) and hybrid (**B**) total knee arthroplasty showing radiolucent lines at the bone–implant interface

Radiographically, according to the Modern Knee Society Radiographic Evaluation System, radiolucent lines, considered potential indicators of implant micromotion, were observed in 18.1% of the hybrid implants (15 of 83) compared to 6.0% of the cementless implants (5 of 83), a significant difference (*p* = 0.032). This corresponded to a relative risk of 0.33 (95% CI, 0.13 to 0.88) for the cementless group. All identified radiolucent lines were thin, non-progressive, and not accompanied by clinical symptoms suggestive of loosening. Implant stability was interpreted in conjunction with the patients’ clinical course during follow-up. These radiographic observations should be interpreted cautiously and should not be considered proof of superior fixation stability or long-term implant survivorship. Representative anteroposterior postoperative radiographs from each fixation group illustrating radiolucent lines are shown in Fig. 2.

As a sensitivity analysis for multiple comparisons, *p*-values for the prespecified family of key secondary postoperative outcomes were additionally adjusted using the Benjamini–Hochberg procedure. These findings remained significant after false discovery rate correction (adjusted *p* = 0.032 for all five outcomes).

### Functional outcomes and mobility

Both groups achieved substantial and similar improvements in knee function. There were no statistically significant differences in postoperative range of motion (ROM), which averaged 97.9° ± 12.4 in the hybrid group vs. 97.9° ± 9.8 in the cementless group (*p* = 0.760). HSS knee scores improved by a mean of 41.15 ± 16.90 points in the hybrid group and 41.12 ± 10.99 in the cementless group, with no significant difference in gain (*p* = 0.599). Final follow-up EQ-5D quality-of-life index was likewise similar (0.84 ± 0.09 hybrid vs. 0.86 ± 0.08 cementless; *p* = 0.104). These findings support comparable mid-term functional recovery and patient-reported outcomes between fixation strategies.

### Propensity-matched analysis

After propensity score matching, 150 patients (75 cementless vs. 75 hybrid) were analyzed with well-balanced baseline characteristics (Table 3). All baseline covariates used for matching (age, sex, BMI, Charlson index, HSS) showed no significant differences between the matched cementless and hybrid sub-cohorts (*p* > 0.8 for all), confirming successful matching.

**Table 3:** Baseline characteristics after propensity score matching (matched sub-cohort, *n* = 150)

Variable	Hybrid (*n* = 75)	Cementless (*n* = 75)	*p*-value
Sex (Male/Female)	38 (50.7%)/37 (49.3%)	38 (50.7%)/37 (49.3%)	1.000
Age (years), mean ± SD	62.0 ± 5.5	61.9 ± 5.3	0.881
BMI (kg/m^2^), mean ± SD	30.3 ± 4.0	30.2 ± 4.1	0.917
Charlson Comorbidity Index	2.3 ± 1.2	2.3 ± 1.2	0.964
Pre-op HSS score, mean ± SD	56.4 ± 12.1	56.7 ± 11.8	0.902

Baseline variables after 1:1 matching show no significant intergroup differences (all *p* > 0.8), indicating successful covariate balance. Matched pairs were formed on age, sex, BMI, Charlson index, and preoperative HSS score

In the matched analysis, the observed patterns from the overall cohort were directionally consistent (Table 4). Cementless TKA was associated with shorter mean operative time than hybrid TKA (66.0 ± 13.0 vs. 77.5 ± 14.0 min, respectively; *p* = 0.044). Postoperative pain reduction on the VAS was also modestly greater in the cementless group (7.3 ± 1.9 vs. 6.5 ± 2.0; *p* = 0.018). Late complications were observed less frequently with cementless fixation (1 of 75 [1.3%] vs. 8 of 75 [10.7%]; *p* = 0.035), and radiolucent lines were observed less often in cementless implants (4 of 75 [5.3%] vs. 13 of 75 [17.3%]; *p* = 0.037). No aseptic loosening occurred in the cementless matched cohort, whereas 2 cases (2.7%) were noted in the matched hybrid cohort (*p* = 0.241), reflecting the low event rate. Importantly, matched cementless and hybrid patients had no significant differences in postoperative ROM or functional score improvements, mirroring the findings of the entire cohort. Overall, the PSM analysis supports the consistency of the observed associations, while not excluding residual confounding from unmeasured factors.

**Table 4:** Clinical outcomes in propensity score-matched cohorts

Outcome	Hybrid (*n* = 75)	Cementless (*n* = 75)	*p*-value
Surgical time (min), mean ± SD	77.5 ± 14.0	66.0 ± 13.0	0.044
VAS pain reduction, mean ± SD	6.5 ± 2.0	7.3 ± 1.9	0.018
Late complications, *n* (%)	8 (10.7%)	1 (1.3%)	0.035
Aseptic loosening, *n* (%)	2 (2.7%)	0 (0.0%)	0.241
Radiolucent lines, *n* (%)	13 (17.3%)	4 (5.3%)	0.037
Postoperative ROM (degrees)	98.1 ± 11.5	97.6 ± 10.2	0.807
HSS score improvement, mean ± SD	40.5 ± 15.0	40.8 ± 11.2	0.912
EQ-5D index (final)	0.85 ± 0.10	0.86 ± 0.09	0.536

Outcomes after matching are directionally consistent with the full cohort analysis. P-values from paired tests (Wilcoxon signed-rank for continuous variables; McNemar’s test for binary outcomes). No significant differences in functional outcomes (ROM, HSS, EQ-5D) were observed, while between-group associations for surgical time, VAS pain reduction, late complications, and radiolucent lines remained directionally consistent

## Discussion

### Summary of main findings

In this retrospective cohort of 166 primary TKAs, cementless and hybrid fixation showed comparable mid-term functional outcomes, quality of life, and range of motion. Pain reduction was statistically greater in the cementless group, although the magnitude of this difference was modest. Aseptic loosening was observed only in the hybrid group and remained infrequent overall, precluding firm conclusions regarding differential fixation durability.

The only clear intraoperative distinction was a shorter surgical time in the cementless group, likely due to the elimination of cement mixing and curing steps. Radiographically, the hybrid group exhibited a higher frequency of small, non-progressive radiolucent lines (18.1% vs. 6.0% in cementless), although these did not correlate with clinical symptoms suggestive of loosening. Overall, these findings support comparable mid-term functional outcomes between cementless and hybrid fixation in appropriately selected patients. Cementless fixation was associated with shorter operative time and lower observed rates of radiolucent lines and late complications. However, given the retrospective design, surgeon-directed allocation, residual confounding by indication, and low absolute number of adverse events, these findings should be interpreted cautiously and should not be considered evidence of definitive superiority of either fixation strategy.

### Comparison with existing literature

Our results align with the prevailing literature on TKA fixation methods. However, most of the available evidence has focused on cementless-versus-cemented TKA rather than on cementless-versus-hybrid fixation. A recent randomized controlled trial by Olson et al. [9] directly compared cemented and cementless TKA using the same implant design with trabecular metal tibial components and reported equivalent patient-reported outcomes and survivorship at 10-year follow-up. In that study, no statistically significant differences were found in Knee Society Scores or Oxford scores, and implant survivorship was comparable between groups (91.5% vs. 95.9%; *p* = 0.60). Importantly, cemented fixation was associated with a higher rate of osteolysis and loosening, possibly due to increased third-body wear. Similarly, meta-analyses have reported comparable outcomes between cementless and cemented TKA. Zhou et al. [10], in a systematic review of RCTs, found no significant differences in implant survival, radiographic outcomes, or clinical scores between the two fixation methods. Franceschetti et al. [11] reviewed young TKA patients (≤ 60 years) and also reported equivalent functional and radiographic results for cemented vs. cementless implants, reinforcing the reliability of modern cementless fixation in high-demand populations. Although these studies do not directly evaluate hybrid fixation, they support the broader concept that contemporary cementless fixation can perform comparably to more conventional strategies in appropriately selected patients.

Our outcome measures mirror those previously reported. In our cohort, postoperative range of motion, HSS improvement, WOMAC, and EQ-5D did not differ significantly between the cementless and hybrid groups, suggesting that the fixation strategy itself may have limited influence on mid-term functional recovery when the same implant family and similar perioperative care are used. By contrast, pain reduction was statistically greater in the cementless group, although the magnitude of this difference was modest and should be interpreted cautiously.

Meanwhile, our finding of a shorter operative time with cementless TKA is in line with prior reports. For example, Yayac et al. [12] reported shorter surgical times and lower operative costs with cementless techniques, attributable to the absence of time spent on cement application and curing. In the present cohort, this difference is best interpreted as an association with operative workflow rather than as evidence of broader clinical superiority; its clinical and economic implications require dedicated evaluation.

Implant loosening events were infrequent in our series. No femoral component loosening was observed in either group, and tibial component loosening was rare. Miller et al. [13] similarly reported low loosening rates with a modern cementless design. However, because the number of loosening events in the present study was very small and follow-up was mid-term, these data cannot support robust conclusions regarding implant survivorship or long-term fixation performance. Radiographically, we observed more non-progressive radiolucent lines in the hybrid group, whereas aseptic loosening events were uncommon overall. Likewise, late complication rates were low in both groups, although observed less frequently in the cementless cohort.

These findings are broadly consistent with meta-analyses by Liu et al. [1] and Zhou et al. [10], who reported no increased risk of infection or revision with cementless fixation. Liu et al. [1] in particular noted equivalent all-cause revision rates and similar failure modes for cemented vs. cementless TKAs, reinforcing that eliminating cement does not inherently compromise the outcome if implants are well-designed and patients are appropriately selected. However, given the very low absolute number of loosening events in our cohort and the use of plain radiographs rather than more sensitive migration analyses, these radiographic differences should be interpreted cautiously and should not be overread as definitive evidence of superior fixation durability.

Notably, most prior studies focused on fully cemented versus cementless comparisons; our study adds data to the less commonly studied hybrid fixation scenario, where literature remains sparse. In addition, hybrid fixation has often been grouped within broader “cemented” categories or not analyzed separately in previous studies, which makes direct comparison difficult and supports the relevance of evaluating it as a distinct strategy. Given the observational design and residual confounding, the present findings should be interpreted as showing comparable mid-term functional outcomes and directionally consistent associations for selected perioperative and radiographic endpoints, rather than as evidence that one fixation strategy is superior to the other.

### Pathophysiological explanations

The comparable outcomes between cementless and hybrid TKA can be understood in light of implant fixation biology. Cementless fixation achieves initial stability through press-fit implantation and promotes long-term stability via osseointegration. The small, non-progressive radiolucent lines we observed in some cementless tibial components likely reflect benign bone remodeling at the bone–implant interface rather than true loosening, especially when these radiolucencies remain < 2 mm and the patient is asymptomatic. By contrast, hybrid fixation uses cement for the tibial component, which can mask early radiographic changes at the bone interface but introduces a mechanical cement layer that is susceptible to fatigue or cement mantle cracks over time. In our cohort, all observed radiolucent lines (in both cementless and hybrid cases) appeared within the first postoperative year, and none showed any progression or expansion on subsequent annual radiographs, underscoring their benign clinical course.

Biomechanically, a well-designed cementless implant offers the potential for biological ingrowth that may result in stronger long-term fixation, whereas cemented implants rely on an interdigitating cement mantle that could degrade under repetitive loading. This distinction is particularly relevant in younger or more obese patients who place greater demands on the prosthesis. Consistent with this concept, Sinicrope et al. [14] found improved loosening-free survivorship with cementless TKA in morbidly obese patients at a minimum 5-year follow-up. However, the present study was not designed to determine long-term fixation mechanisms or survivorship. Therefore, the absence of early loosening in the cementless cohort should be interpreted in the context of patient selection, limited follow-up, and low event counts, rather than as proof of superior long-term fixation. Previous studies, including that by Wang et al. [15], have reported favorable findings for cementless fixation in younger patients, but these data should be considered supportive background rather than direct evidence that cementless fixation provides superior outcomes in the present cohort.

Bone quality remains a critical factor in choosing the fixation method. Cementless implants require sufficient bone stock for initial press-fit stability and subsequent ingrowth, making hybrid or fully cemented fixation more appropriate when tibial bone quality or press-fit stability is considered insufficient. In our cohort, fixation type was selected using surgeon-directed intraoperative assessment rather than a standardized objective bone-quality measure, and this selection process may have contributed to the observed between-group differences. In selected patients, cementless implants are intended to permit osseointegration, whereas cementing the tibial component in hybrid cases served as a pragmatic strategy when press-fit stability was less certain.

Advancements in implant design may partly explain the renewed interest in cementless fixation. Highly porous coatings and modern tibial baseplate designs are intended to improve initial stability and support biological fixation. Miller et al. [13] reported low early loosening rates with a modern cementless TKA design, providing relevant background for the interpretation of contemporary cementless fixation. However, the present study was not designed to directly evaluate osseointegration, implant migration, or fixation mechanics. Therefore, any interpretation regarding implant design should be considered contextual rather than mechanistic, and the observed radiographic findings should not be taken as evidence of superior long-term fixation performance.

### Clinical implications

Our findings suggest that cementless TKA may represent a reasonable alternative to hybrid fixation in appropriately selected patients. When bone quality is considered adequate—such as in younger or active older patients—cementless fixation was associated with similar functional recovery and quality of life, together with shorter operative time and lower observed rates of radiolucent lines and late complications. However, given the retrospective design, surgeon-directed allocation, residual confounding, and low absolute number of adverse events, these observations should be considered hypothesis-generating and should not be interpreted as definitive evidence of superiority. In practice, these results suggest that fully cementless TKA may be considered in eligible patients without clear evidence of worse mid-term functional outcomes than hybrid fixation.

Additionally, the shorter operative time we observed with cementless TKA, approximately 12 min shorter on average, may offer advantages for surgical efficiency. A reduced tourniquet time and quicker procedure can improve operating room turnover and may decrease anesthesia duration for patients. From a cost perspective, time savings could offset the typically higher implant cost of cementless components, potentially making the overall approach cost-neutral; prior economic analyses have suggested as much [12]. Moreover, in revision situations, using a cementless primary implant may simplify the procedure by avoiding the need for cement removal and preserving bone stock.

Importantly, we found no increase in infection risk in the cementless group despite the lack of antibiotic-laden cement, which aligns with prior reports that systemic antibiotic prophylaxis remains effective in cementless arthroplasty when appropriately implemented [1]. Within the limitations of this retrospective cohort, the comparable functional outcomes and the absence of an apparent safety disadvantage in the cementless group suggest that, when modern implants are used in appropriately selected patients, cementless fixation does not appear to compromise mid-term outcomes relative to hybrid fixation.

### Study limitations

This study has several limitations inherent to its retrospective, single-center design. Most importantly, fixation type was not randomly assigned but was determined by the operating surgeons according to intraoperative assessment of tibial bone stock and press-fit stability. Because this assessment was not standardized or quantified with an objective measure such as DEXA/BMD or a prespecified intraoperative scoring system, persistent confounding by indication is likely. Although strict inclusion criteria, baseline comparability, propensity score matching, and multivariable adjustment were used to mitigate measured confounding, these methods cannot account for unmeasured variables, including patient activity level, deformity severity within the included range, subtle bone-quality differences, temporal adoption effects, and surgeon decision factors. Therefore, observed differences between fixation groups should be interpreted as associations rather than treatment effects.

The relatively modest sample size and mid-term follow-up (mean approximately 3 years) limit the ability to detect very rare complications or longer-term differences in outcomes such as polyethylene wear, osteolysis, or late loosening that might emerge beyond the timeframe of this study. The study was underpowered for rare outcomes, including aseptic loosening and late complications, and the observed differences may reflect random variation rather than a true effect. These findings should therefore be considered hypothesis-generating and interpreted with caution. The statistically significant difference in pain reduction should likewise be interpreted conservatively, given its modest magnitude. Similarly, the absence of statistically significant differences in some postoperative functional outcomes may reflect either true comparability between fixation strategies or limited power to detect smaller between-group differences. Although a sensitivity analysis using the Benjamini–Hochberg procedure was performed and the main secondary findings remained significant after false discovery rate correction, these outcomes should still be interpreted cautiously given the observational design and the possibility of residual confounding.

Selective patellar resurfacing was performed according to intraoperative cartilage assessment and surgeon judgment rather than a rigid pre-specified algorithm, which may have introduced additional treatment heterogeneity. Individual complication subtypes were too infrequent to support robust category-specific comparative analysis. Radiographic assessment was based on standard X-rays; we did not use advanced imaging techniques such as radiostereometric analysis (RSA), which could detect micro-motion or migration with greater sensitivity. Implant stability was assessed primarily through conventional radiographic follow-up and clinical course rather than a dedicated standardized clinical stability protocol. Accordingly, radiographic differences in non-progressive radiolucent lines should not be overinterpreted as evidence of superior fixation performance or long-term implant stability.

Additionally, our results pertain to a specific implant design (Triathlon® CR) and a cohort of patients carefully selected for cementless suitability; thus, generalizability to other implant designs, older patients, or less experienced surgical settings may be limited. All surgeries were performed by an experienced team of senior arthroplasty surgeons rather than by a single surgeon, which may reduce but does not eliminate the possibility of surgeon-dependent effects on implant selection and outcomes. Accordingly, our findings should not be interpreted as evidence of long-term superiority or definitive survivorship differences between fixation strategies.

### Future research directions

Future investigations should include well-powered, preferably randomized trials directly comparing cementless, hybrid, and fully cemented TKA across diverse patient populations. Extended follow-up beyond 10 years will be crucial to evaluate long-term implant survivorship and to reveal any delayed advantages or failure modes associated with each fixation method. Such studies should incorporate objective bone-quality assessment, standardized criteria for fixation selection, and sufficient sample sizes to evaluate rare outcomes such as aseptic loosening and late complications.

Subgroup analyses are needed to clarify whether specific patient groups may be better suited to one fixation strategy or another—for instance, whether elderly patients or those with osteoporotic bone have more predictable outcomes with cemented tibial fixation, or whether younger, high-demand patients may derive durable benefit from cementless fixation over longer follow-up. The role of emerging surgical innovations, such as robotic-assisted TKA, in optimizing component alignment and press-fit technique also warrants further investigation, although their potential impact on fixation-related outcomes should be evaluated in dedicated prospective studies.

Moreover, the use of advanced imaging modalities and implant tracking could enhance our understanding of the osseointegration process and the significance of radiolucent lines. Retrieval studies of failed components (both cementless and hybrid) may shed light on the quality of biological fixation achieved. Finally, large-scale joint registries and meta-analyses will be valuable for identifying long-term trends in fixation choice, outcomes, and cost-effectiveness, ultimately guiding best practices in primary TKA fixation strategy.

## Conclusions

In this retrospective cohort study comparing cementless and hybrid fixation, cementless TKA was associated with shorter operative time and lower observed rates of radiolucent lines and late complications, while functional outcomes, quality of life, and postoperative mobility were comparable between groups. The statistically significant difference in pain reduction was modest, and the very low number of loosening and late complication events means that these rare-outcome findings are underpowered, statistically unstable, and hypothesis-generating.

These findings suggest that cementless fixation may represent a reasonable alternative to hybrid fixation in appropriately selected patients with good bone quality. However, because of the retrospective design, surgeon-directed allocation, persistent residual confounding, low event counts, and mid-term follow-up, the present study supports association-based observations only and should not be interpreted as demonstrating definitive superiority of cementless fixation. Confirmation in longer-term prospective comparative studies is required.

## Data Availability

The datasets used and/or analyzed during the current study are available from the corresponding author on reasonable request.

## References

[1] Liu Y, Zeng Y, Wu Y, Li M, Xie H, Shen B. A comprehensive comparison between cementless and cemented fixation in the total knee arthroplasty: an updated systematic review and meta-analysis. J Orthop Surg Res. 2021;16(1):176. 10.1186/s13018-021-02299-4. 33673850 10.1186/s13018-021-02299-4 PMC7934367

[2] Meneghini R, Hanssen A. Cementless fixation in total knee arthroplasty: past, present, and future. J Knee Surg. 2008;21(4):307–14. 10.1055/s-0030-1247837. 18979934 10.1055/s-0030-1247837

[3] Zingg M, Miozzari HH, Fritschy D, Hoffmeyer P, Lübbeke A. Influence of body mass index on revision rates after primary total knee arthroplasty. Int Orthop. 2016;40(4):723–9. 10.1007/s00264-015-3031-0. 26559943 10.1007/s00264-015-3031-0

[4] Aggarwal VK, Goyal N, Deirmengian G, Rangavajulla A, Parvizi J, Austin MS. Revision total knee arthroplasty in the young patient: is there trouble on the horizon? J Bone Joint Surg Am. 2014;96(7):536–42. 10.2106/JBJS.M.00131. 24695919 10.2106/JBJS.M.00131

[5] Walker-Santiago R, Tegethoff JD, Ralston WM, Keeney JA. Revision total knee arthroplasty in young patients: higher early reoperation and rerevision. J Arthroplasty. 2021;36(2):653–6. 10.1016/j.arth.2020.08.052. 32948426 10.1016/j.arth.2020.08.052

[6] Roche M, Law T, Kurowicki J, Rosas S, Rush A. Effect of obesity on total knee arthroplasty costs and revision rate. J Knee Surg. 2018;31(1):38–42. 10.1055/s-0037-1608933. 29216676 10.1055/s-0037-1608933 PMC6427916

[7] Prasad AK, Tan JHS, Bedair HS, Dawson-Bowling S, Hanna SA. Cemented vs. cementless fixation in primary total knee arthroplasty: a systematic review and meta-analysis. EFORT Open Rev. 2020;5(11):793–8. 10.1302/2058-5241.5.200030. 33312706 10.1302/2058-5241.5.200030 PMC7722941

[8] Matassi F, Carulli C, Civinini R, Innocenti M. Cemented versus cementless fixation in total knee arthroplasty. Joints. 2013;1(3):121–5 PMID:25606521. 25606521 PMC4295702

[9] Olson NR, Parks NL, Nagda SS, McAsey CJ, Fricka KB. To cement or not? Ten-Year results of a prospective, randomized study comparing cemented versus cementless total knee arthroplasty. J Arthroplasty. 2025. 10.1016/j.arth.2025.04.076. 40339944 10.1016/j.arth.2025.04.076

[10] Zhou K, Yu H, Li J, Wang H, Zhou Z, Pei F. No difference in implant survivorship and clinical outcomes between full-cementless and full-cemented fixation in primary total knee arthroplasty: a systematic review and meta-analysis. Int J Surg. 2018;53:312–9. 10.1016/j.ijsu.2018.04.015. 29656129 10.1016/j.ijsu.2018.04.015

[11] Franceschetti E, Torre G, Palumbo A, Papalia R, Paciotti M, Maffulli N, et al. No difference between cemented and cementless total knee arthroplasty in young patients: a review of the evidence. Open Orthop J. 2017;11:133–8. 10.2174/1874325001711010133. 28332044 10.1007/s00167-017-4519-5

[12] Yayac M, Harrer S, Hozack WJ. Cementless total knee arthroplasty: a resurgence—who, when, and how. J Arthroplasty. 2024;39(1):S45–53. 10.1016/j.arth.2023.08.019. 38458333 10.1016/j.arth.2024.02.078

[13] Miller AJ, Stimac JD, Smith LS, Feher AW, Bhimani SJ, Mont MA. Results of cemented vs cementless primary total knee arthroplasty using the same implant design. J Arthroplasty. 2018;33(4):1089–93. 10.1016/j.arth.2017.11.048. 29275115 10.1016/j.arth.2017.11.048

[14] Sinicrope BJ, Feher AW, Bhimani SJ, Smith LS, Harwin SF, Yakkanti MR, et al. Increased survivorship of cementless versus cemented TKA in the morbidly obese. A minimum 5-year follow-up. J Arthroplasty. 2019;34(2):309–14. 10.1016/j.arth.2018.10.016. 30446183 10.1016/j.arth.2018.10.016

[15] Wang K, Sun H, Zhang K, Li S, Wu G, Zhou J, et al. Better outcomes are associated with cementless fixation in primary total knee arthroplasty in young patients: a systematic review and meta-analysis of randomized controlled trials. Medicine (Baltimore). 2020;99(3):e18750. 10.1097/MD.0000000000018750. 32011458 10.1097/MD.0000000000018750 PMC7220050

